# Effect of physical activity on telomere length among middle-aged Indian adults: A cross-sectional study

**DOI:** 10.6026/973206300221627

**Published:** 2026-03-31

**Authors:** Meena Mirdha, Samanvithaa Regalla

**Affiliations:** 1 Department of Physiology, AIIMS, Bathinda, Punjab, India; 2 Department of Physiology, Kamineni Academy of Medical Sciences and Research Center, L.B Nagar Hyderabad, India

**Keywords:** Aging, biological aging, physical activity, telomere length, middle-aged adults

## Abstract

A general problem in aging research understands the factors that influence biological aging, particularly the role of lifestyle behaviors such as physical activity. Therefore, it is of interest to examine the relationship between physical activity levels and leukocyte telomere length among middle-aged adults. Thus, we show the potential protective role of physical activity in maintaining cellular health during midlife. This research advances knowledge by establishing a significant link between higher physical activity levels and longer telomere length in middle-aged adults.

## Background:

Aging is a complex, multifactorial biological process characterized by a gradual decline in physiological function and an increased susceptibility to chronic diseases. While chronological age is commonly used to describe aging, it does not fully capture the variability in health status observed among individuals of the same age [1]. This has led to growing interest in biological markers of aging that more accurately reflect cellular and molecular changes occurring over time. Among these biomarkers, telomere length has emerged as a key indicator of cellular aging and overall biological age [2]. Telomeres are repetitive nucleotide sequences (TTAGGG) located at the ends of linear chromosomes, where they serve a critical protective role by maintaining chromosomal stability and preventing end-to-end fusion. With each cell division, telomeres progressively shorten due to the end-replication problem and limited activity of the enzyme telomerase in most somatic cells [3]. Excessive telomere shortening can trigger cellular senescence or apoptosis, contributing to tissue dysfunction and the development of age-related diseases. Shorter telomere length has been associated with a variety of adverse health outcomes, including cardiovascular disease, type 2 diabetes mellitus, cancer and increased all-cause mortality [4]. In addition to genetic determinants, telomere length is strongly influenced by environmental and lifestyle factors. Oxidative stress and chronic inflammation are known to accelerate telomere attrition by inducing DNA damage and impairing telomere repair mechanisms. Conversely, lifestyle behaviors that reduce oxidative stress and inflammation may help preserve telomere length [5]. Factors such as diet quality, psychological stress, sleep patterns, smoking and physical activity have therefore been increasingly investigated for their potential role in modulating telomere dynamics [6]. Physical activity is widely recognized as a cornerstone of healthy aging. Regular engagement in physical activity has been shown to improve cardiovascular fitness, metabolic health, musculoskeletal strength and mental well-being, while reducing the risk of non-communicable diseases and premature mortality [7]. At the cellular level, physical activity has been proposed to exert anti-aging effects by enhancing antioxidant defense systems, improving mitochondrial function, reducing systemic inflammation and up regulating telomerase activity. These biological mechanisms suggest that physically active individuals may experience slower telomere shortening compared to their sedentary counterparts [8].

Despite these proposed benefits, existing evidence on the association between physical activity and telomere length remains mixed. Several observational studies have reported longer telomeres among individuals engaging in moderate to vigorous physical activity, supporting a protective relationship [9]. In contrast, other studies have found weak or no associations, or have suggested that only specific intensities or durations of physical activity confer telomere-related benefits. These inconsistencies may be attributed to differences in study populations, age ranges, physical activity assessment methods, laboratory techniques for telomere measurement and the extent to which confounding factors were controlled [10]. Middle-aged adulthood represents a particularly important yet understudied life stage in telomere research. This period, typically spanning the ages of 40 to 60 years, is marked by the early onset of many chronic diseases and accelerated biological aging processes [11]. Importantly, lifestyle habits during middle age can have long-lasting effects on health outcomes in later life. Studying telomere length in this age group provides a valuable opportunity to identify modifiable behaviors that may slow cellular aging before irreversible damage occurs. Physical activity interventions initiated during middle age may therefore have significant implications for extending health span and reducing disease burden [12]. Furthermore, cross-sectional studies examining physical activity and telomere length offer practical insights into real-world associations within community-dwelling populations. While longitudinal studies are ideal for establishing causality, cross-sectional designs remain essential for generating hypotheses, identifying population-level trends and guiding public health strategies [13]. By evaluating telomere length across different levels of physical activity, such studies can help clarify whether physically active lifestyles are associated with more favorable cellular aging profiles in middle-aged adults [14]. Given the rising prevalence of sedentary behavior and age-related chronic conditions worldwide, understanding the relationship between physical activity and telomere biology is of considerable public health relevance. Establishing an association between higher physical activity levels and longer telomere length would strengthen the biological rationale for promoting physical activity not only as a means of disease prevention but also as a strategy to delay cellular aging [15]. Therefore, it is of interest to determine the association between physical activity levels and telomere length in middle-aged adults and to assess whether physical activity may serve as a protective factor against accelerated biological aging.

## Materials and Methods

## Study design and study setting:

This original research was conducted as a cross-sectional observational study to evaluate the association between physical activity levels and telomere length among middle-aged adults. The study was carried out in a community-based setting over a period of six months after obtaining approval from the Institutional Ethics Committee. All procedures were performed in accordance with the ethical standards laid down in the Declaration of Helsinki.

## Study population and sample size:

A total of 100 middle-aged adults aged between 40 and 60 years were included in the study. The sample size was determined based on feasibility and previous similar studies assessing telomere length and lifestyle factors, with an assumed moderate effect size and a confidence level of 95%. Participants were recruited using convenience sampling from the general population through health camps and outpatient clinics.

## Inclusion criteria:

Participants who met the following criteria were included in the study:

[1] Adults aged 40-60 years

[2] Both males and females

[3] Apparently healthy individuals willing to provide written informed consent

[4] Individuals able to complete the physical activity questionnaire

## Exclusion criteria:

Participants were excluded if they had:

[1] A history of malignancy, autoimmune disorders, or chronic inflammatory diseases

[2] Acute or chronic infections at the time of sample collection

[3] Long-term use of corticosteroids or immunosuppressive drugs

[4] Current pregnancy

[5] History of genetic disorders known to affect telomere biology

## Assessment of physical activity:

Physical activity levels were assessed using a validated standardized physical activity questionnaire. Participants were categorized into three groups based on their total physical activity scores:

[1] Low physical activity

[2] Moderate physical activity

[3] High physical activity

The questionnaire captured information on frequency, duration and intensity of physical activity performed during work, transportation, household activities and leisure time.

## Collection of demographic and clinical data:

A structured proforma was used to record demographic details such as age and sex, along with lifestyle-related factors including smoking status and alcohol consumption. Anthropometric measurements including height and weight were recorded using standardized techniques and body mass index (BMI) was calculated as weight in kilograms divided by height in meters squared (kg/m^2^).

## Blood sample collection and telomere length measurement:

Venous blood samples (5 mL) were collected under aseptic conditions into EDTA-coated vacutainers. Genomic DNA was extracted from peripheral blood leukocytes using a commercially available DNA extraction kit according to the manufacturer's instructions. Telomere length was measured using quantitative real-time polymerase chain reaction (qPCR), expressed as the telomere-to-single-copy gene (T/S) ratio, which reflects relative telomere length.

## Quality control measures:

All samples were analyzed in duplicate to ensure reliability. Inter-assay and intra-assay coefficients of variation were maintained within acceptable limits. Laboratory personnel were blinded to the physical activity status of participants to minimize measurement bias.

## Statistical analysis:

Data were entered into Microsoft Excel and analyzed using Statistical Package for the Social Sciences (SPSS) version 25.0. Continuous variables were expressed as mean ± standard deviation, while categorical variables were presented as frequencies and percentages. Differences in mean telomere length across physical activity groups were analyzed using one-way analysis of variance (ANOVA). Multivariable linear regression analysis was performed to assess the association between physical activity levels and telomere length after adjusting for potential confounders such as age, sex, BMI and smoking status. A p-value of <0.05 was considered statistically significant.

## Ethical considerations:

The study was conducted after obtaining approval from the Institutional Ethics Committee and in accordance with the Declaration of Helsinki. Written informed consent was obtained from all participants prior to enrollment. Participation was voluntary and confidentiality of personal information was strictly maintained using coded data. Blood samples were collected under aseptic conditions by trained personnel and all data were used solely for research purposes.

## Results:

A total of 100 middle-aged adults (40-60 years) were included in the final analysis. Participants were categorized into low, moderate and high physical activity groups based on questionnaire scores. The results are presented below with corresponding tables, which are cited in the text. Table 1 (see PDF) shows the baseline demographic and clinical characteristics of the participants. The mean age of the study population was 49.8 ± 5.6 years, with a nearly equal distribution of males and females. As shown in Table 2 (see PDF), 34% of participants reported low physical activity, while 36% and 30% were categorized as having moderate and high physical activity levels, respectively. Table 3 (see PDF) demonstrates a statistically significant increase in mean telomere length with higher levels of physical activity (p < 0.001). Participants with high physical activity exhibited the longest telomeres, followed by those with moderate and low activity levels. Table 4 (see PDF) presents the multivariable linear regression analysis generated using STATA. After adjusting for age, BMI and smoking status, both moderate and high physical activity levels remained significantly associated with longer telomere length, with high physical activity showing the strongest positive association. As shown in Table 5 (see PDF), a moderate positive correlation was observed between physical activity score and telomere length (r = 0.46, p < 0.001), indicating that higher physical activity levels were associated with longer telomeres.

## Discussion:

In this study of 100 middle-aged adults, we observed a clear positive association between higher levels of physical activity and longer leukocyte telomere length, with physically active individuals showing significantly greater telomere length even after adjusting for confounders such as age, BMI and smoking. These findings align with and expand upon the existing literature that has explored the relationship between physical activity and telomere dynamics across different populations. Consistent with our results, Tucker *et al.* (2017) [16] found that adults with higher levels of physical activity exhibited longer telomeres in a large cross-sectional analysis of the National Health and Nutrition Examination Survey (NHANES), indicating a potential "biological age advantage" of several years in highly active versus sedentary individuals. Their results suggested that high physical activity was significantly and meaningfully associated with longer leukocyte telomere length, supporting the notion that physical activity may contribute to slower cellular aging. Our findings also resonate with results from broader meta-analyses. For example, Valente *et al.* (2021) [17] reported that physically active individuals tended to have longer telomeres compared to inactive controls, with the association being particularly notable in middle-aged cohorts. Although the overall certainty of evidence was graded low due to heterogeneity across studies, the consistent trend across multiple observational analyses reinforces the idea that physical activity relates positively to telomere length. However, the literature also highlights complexities in this field. Savela *et al.* (2013) [18] in a long-term cohort study observed that moderate physical activity in midlife was associated with longer telomere length measured decades later in older age, whereas high physical activity showed no additional telomere benefits. This suggests that the relationship between activity level, duration and biological aging may not be strictly linear and that moderate, sustained activity might confer optimal benefits. Similarly, findings from the Oulu Cohort (Stenbäck *et al.* 2019) [19] indicated that total steps and reduced sedentary time correlated with telomere length in elderly adults, with some gender-specific variations in this association. While that study focused on older rather than middle-aged populations, the directional consistency (higher activity corresponding with longer telomeres) supports our cross-sectional observations. In addition to observational studies, broader systematic reviews such as Arsenis *et al.* (2017) [20] have discussed potential biological mechanisms that underlie the observed associations, noting that physical activity can modulate oxidative stress, inflammation and telomerase activity all of which influence telomere maintenance. Their review concluded that while higher physical activity levels are generally related to longer telomeres, variability in study design and measurement methods highlights the need for more standardized research, particularly in middle-aged and older adult populations. Taken together, our study adds to this body of evidence by confirming that increased physical activity is associated with longer telomeres in a middle-aged cohort and that this relationship persists after statistical adjustment for key demographic and lifestyle factors. Our results complement national survey data, long-term cohort analyses and meta-analytic evidence, emphasizing that physical activity may serve as a modifiable lifestyle factor influencing biological aging processes. However, the mixed findings from some longitudinal and interventional studies underscore that the association between activity intensity, duration and telomere outcomes can be complex. Factors such as the type of activity, the measurement of physical activity (self-reported versus objective), genetic variability and co-existing health conditions may all contribute to differential effects on telomere length. Further longitudinal and mechanistic studies are needed to clarify these nuances and to establish causal pathways.

## Conclusion:

We show a significant positive association between physical activity levels and telomere length in middle-aged adults. Individuals with moderate to high physical activity had longer telomeres, suggesting a protective role against biological aging. Promoting an active lifestyle in midlife may improve long-term health, though further studies are needed to confirm causality.
